# Objections to Amalgam

**Published:** 1894-06

**Authors:** 


ARTICLE VI.

### OBJECTIONS TO AMALGAM.

Dr. C. Edmun Kells says, in April *Cosmos:* "One of the objections, and without doubt the most serious one to the use of amalgam, is that perfectly uniform results are not obtained."

Is this an objection to amalgam only? Does it not obtain equal force against all other filling materials? Why single out amalgam? We are all perplexed sometimes with the action of cement and gutta percha, and even with gold,—"even in the same mouth,"

This is the reason we are all so careful in selecting our filling materials, and it is the reason why the best are so generally in use by our best dentists, though the flood wood of the profession will take anything that is the cheapest. This is the reason, too, that our most prominent, skilful and experienced manufacturers have to be exceedingly and continually careful in every stage of their preparations; and that the man who is the most successful has his fortune, though he may have but little profit on each package. Dentists are now too intelligent to be deceived by flaming advertisements or mysterious assumptions. As Prof. Shepard, of Boston once said at a clinic in Philadelphia, when some one handed him a new preparation of gold for filling, and essayed to tell him its superior manipulative qualities, "Put it on the tray, and I will soon tell you what it is. The results will tell.

In the manufacture of alloy the manipulation is quite as important as the materials used. With many it is difficult to melt tin with silver, without burning the tin, and this is still more difficult when gold and platina are added, and also to have the mix uniform. But experience overcomes these difficulties.

Dr. Kells says "different lots of the same brand of alloy may and do vary in composition." Certainly; but is this an unfathomable mystery? Is it an inexplicable hap? While there are makes that do vary in quality, there are others which do not, because made invariably the same and by the same master hand, and in the same heat.

Then again, there must be skill in the selection of materials. Some years ago we were offered some Western bricks of silver below the market price of our alloy. We found lead in it. Suppose we had used it because it was cheap? We should have been as impolitic as a dentist who wrote us "I have been using your alloy for years with uniform success till lately. I bought some from a Western manufacturer, who warranted it to be the same, or at least as good as your own make, and I cannot use it with satisfaction."

At another time our merchant sent us a pig of tin which had lead in its composition. When expostulated with he said, "Oh, I can't believe the little lead in that tin can make any difference." But that little cost me fifty dollars to eliminate from a single batch.

Yes, and we know that without much experience and careful personal supervision in making alloy, "it is practically impossible to manipulate it in a uniform manner, and under the same conditions." But no more so than with other filling materials, and with all of them the manipulation of the dentist in using it is quite as important as the manipulation in the making. When we see the large number of jacks and cobblers using alloy so bunglingly and ignorantly, we are astonished, not "that there are perfectly uniform results," but that in spite of these empires "this much-abused material" does so well. If half the intelligence, care and skill were used with alloy—even by the best operators—as is bestowed on gold, we should soon find that alloy is the best material for filling teeth, except where its color is conspicuous.

Dr. Kells is also right in saying of the making of alloy: "The process of crystallization may produce varying resultant masses, as it, itself, has undergone under varying conditions." But that manufacturer will have to step down and out who cannot control this crystallization, and that dentist who cannot so talk to his alloy as to have it crystallize under the most favorable conditions, is not a magician. The custom of some dentists in mixing up a mass sufficient for a dozen fillings and expecting the last to be as good as the first, is an ignoramus. Yet we have seen dentists who use a mass that is very soft, so it will last for several fillings, and when, even then, it becomes crumbly, add more murcury, expecting it to be "just as good."—*Editorial in Items of Interest.*

